# Supplementary material for: Voltage-Gated Sodium Channel NaV1.5 Controls NHE−1−Dependent Invasive Properties in Colon Cancer Cells
Source: Cancers (Basel). 2022 Dec 22;15(1):46. doi: 10.3390/cancers15010046 (PMC9817685; doi:10.3390/cancers15010046)
Supplement: Supplementary file 1 [file cancers-15-00046-s001.zip › Figure S1 Exploration of the epithelial phenotype of tumor-derived primary cells.pdf]

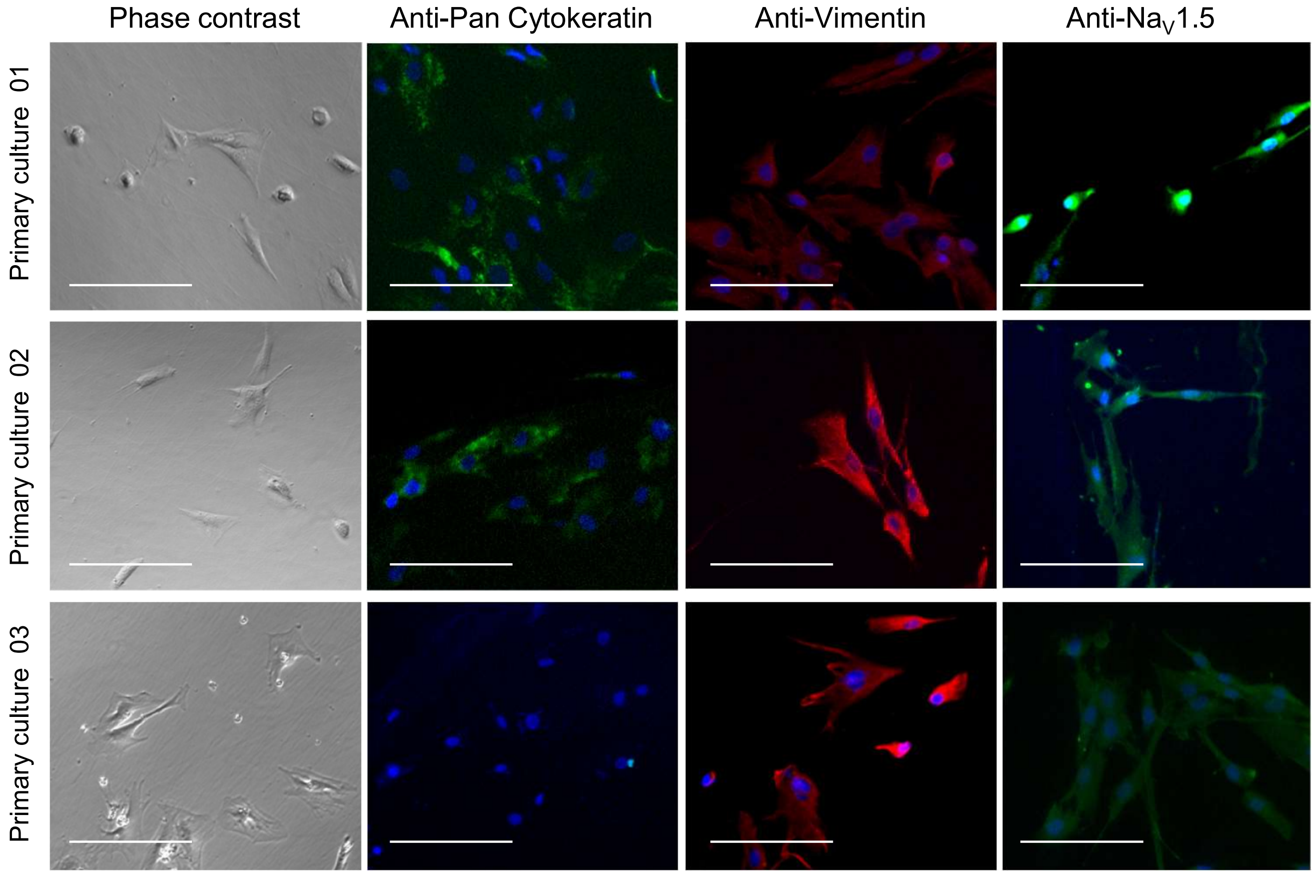

Figure S1. Exploration of the epithelial (cytokeratin) and mesenchymal (vimentin) markers in tumor derived primary cells. Columns of the panel show phase contrast and epifluorescence microscopy images for the protein cytokeratin (green, detected with the primary antibody Purified anti-Cytokeratin (pan reactive) BioLegend (San Diego, CA) Ref. 628602), Vimentin (red, detected with the primary antibody Purified anti-Vimentin BioLegend (San Diego, CA) Ref. 699302) and for the sodium channel NaV1.5 (green, detected with the Anti-Sodium Channel Na$_V$1.5 antibody produced in rabbit Sigma-Aldrich Ref. S0819) from three primary cultures obtained from tumor-derived cells. Scale bar 70 μm.
