# Supplementary material for: Voltage-Gated Sodium Channel NaV1.5 Controls NHE−1−Dependent Invasive Properties in Colon Cancer Cells
Source: Cancers (Basel). 2022 Dec 22;15(1):46. doi: 10.3390/cancers15010046 (PMC9817685; doi:10.3390/cancers15010046)
Supplement: Supplementary file 1 [file cancers-15-00046-s001.zip › Figure S3 Detection and colocalization analysis of Nav1.5 and NHE-1 in colon cancer cells with primary antibody pair No. 2.pdf]

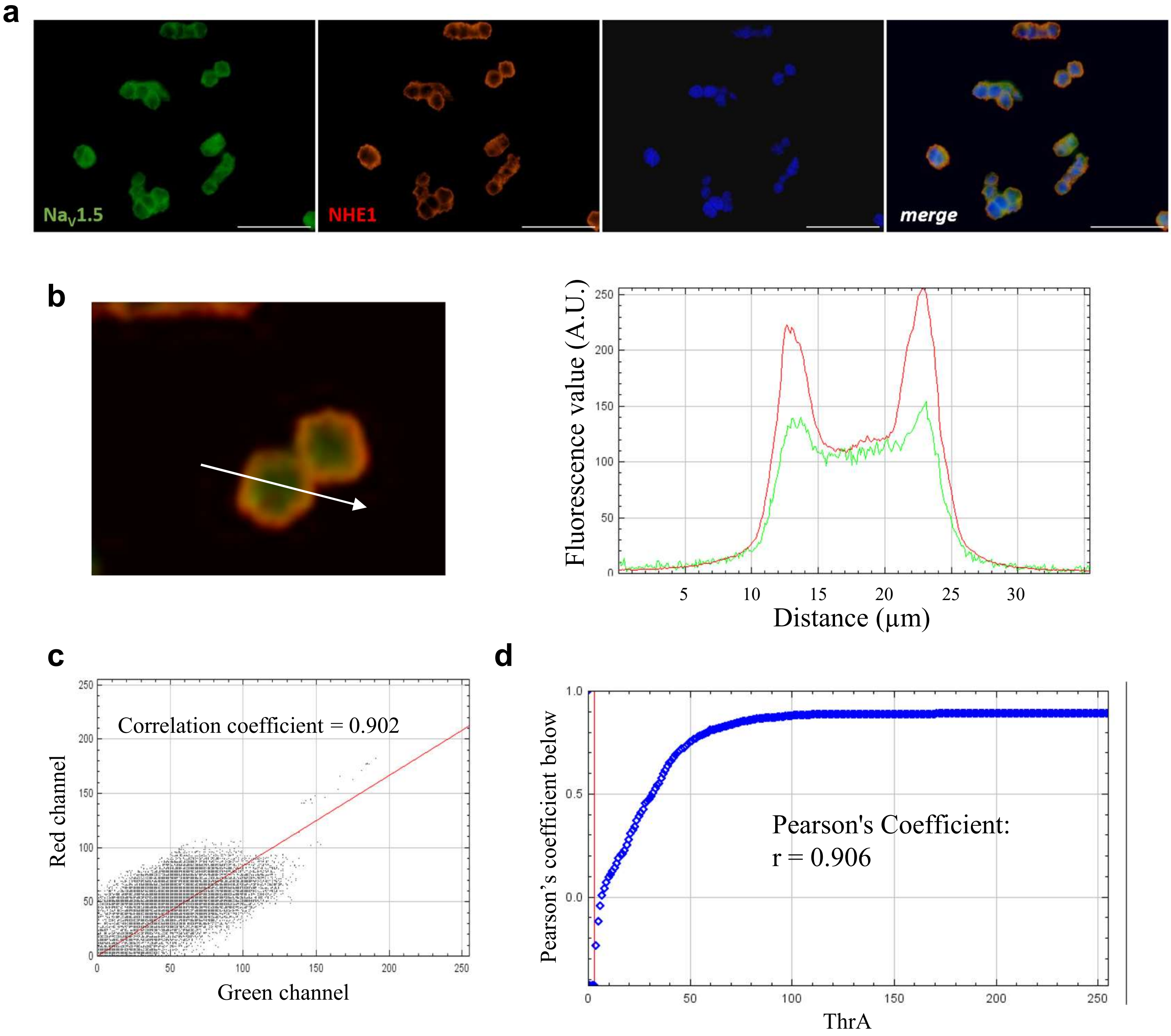

Figure S3. Detection and colocalization analysis of $Na_V1.5$ and NHE-1 in colon cancer cells with primary antibody pair No. 2. (a) Epifluorescence microscopy analysis of $Na_V1.5$ and NHE-1 proteins in SW620 cells. Images show the staining for $Na_V1.5$ proteins (green, detected with the Anti-$Na_V1.5$ Na+ CP Vα Santa Cruz Biotechnologies Ref. sc-271255), NHE-1 (red, detected with the Anti-NHE-1 antibody Abcam Ref. ab67314), nuclei (blue, DAPI) and the merge image. Both proteins $Na_V1.5$ and NHE-1 are located at the plasma membrane of cancer cells. **(b)** A representative example of merging the green and red channels of the epifluorescence images is shown. The right panel shows the fluorescence profile for the two channels along the linear segmentation indicated by the white arrow. the arrow indicates a segmentation taken for fluorescence profile analysis. **(c)** Cytofluorogram for the fluorescence signals and spatial distribution corresponding to $Na_V1.5$ and NHE-1 proteins from images showed in (a). **(d)** Plot shows the Costes' automatic threshold analysis which takes into account that the limiting values of each channel are initialized with the maximum intensity of each channel and are progressively decreased. Pearson's coefficient is calculated at the same time for each increment. Pearson' coefficient obtained was r = 0.906. Scale bar 50 μm.
