# Supplementary material for: Voltage-Gated Sodium Channel NaV1.5 Controls NHE−1−Dependent Invasive Properties in Colon Cancer Cells
Source: Cancers (Basel). 2022 Dec 22;15(1):46. doi: 10.3390/cancers15010046 (PMC9817685; doi:10.3390/cancers15010046)
Supplement: Supplementary file 1 [file cancers-15-00046-s001.zip › Figure S4 In silico analysis for the prediction of NHE-1-Nav1.5 proteins interaction.pdf]

a

P19634 · SL9A1_HUMAN P19634-1
MVLRSGICGLSPHRIFPSLLVVVALVGLLPVLRSHGLQLSPTASTIRSSEPPRERSIGDVTTAPPEVTPESRPVNHSVTDHGMKPRKAFPVLGIDYTHVRTPFEISLWILLACLMKIGFHVIPTISSIVPESCLLIVVGLLVGGLIKGVGETPPFLQSDVFFLFLLPPIILDAGYFLPLRQFTENLG
TILIFAVVGTLWNAFFLGGLMYAVCLVGGEQINNIGLLDNLLFGSIISAVDPVAVLAVFEEIHINELLHILVFGESLLNDAVTVVLYHLFEEFANYEHVGIVDIFLGFLSFFVVALGGVLVGVVYGVIAAFTSRFTSHIRVIEPLFVFLYSYMAYLSAELFHLSGIMALIASGVVMRPYVEANIS
HKSHTTIKYFLKMWSSVSETLIFIFLGVSTVAGSHHWNWTFVISTLLFCLIARVLGVLGLTWFINKFRIVKLTPKDQFIIAYGGLRGAIAFSLGYLLDKKHFPMCDLFLTAIITVIFFTVFVQGMTIRPLVDLLAVKKKQETKRSINEEIHTQFLDHLLTGIEDICGHYGHHHWKDKLNRFNKK
YVKKCLIAGERSKEPQLIAFYHKMEMKQAIELVESGGMGKIPSAVSTVSMQNIHPKSLPSERILPALSKDKEEEIRKILRNNLQKTRQRLRSYNRHTLVADPYEEAWNQMLLRRQKARQLEQKINNYLTVPAHKLDSPTMSRARIGSDPLAYEPKEDLPVITIDPASPQSPESVDLVNE
ELKGKVLGLSRDPAKVAEEDEDDDGGIMMRSKETSSPGTDDVFTPAPSDSPSSQRIQRCLSDPGPHPEPGEGEPFFPKGQMVSGDRIHCMDILFAFTKRVLGESGEMDALKIQMEEKFMAANPSKISYEPITTTLRRKHEEVSAMVIQRAFRRHLLQRSLKHASFLFRQQAGSGLS
EEDAPEREGLIAYVMSENFSRPLGPPSSSSISSTSFPPSYDSVTRATSDNLQVRGSDYSHSEDLADFPPSPDRDRESIV

Q14524 · SCN5A_HUMAN Q14524-1
MANFLLPRGTSSFRRFTRESLAAIEKRMAEKQARGSTTLQESREGLPEEEAPRPQLDLQASKKLPDLYGNPPQELIGEPLEDLDPFYSTQKTFIVLNKGKTIFRFSATNALYVLSPFHPIRRAAVKILVHSLFNMLIMCTILTNCVFMAQHDPPPWTKYVEYTFTAIYTFESLVKILARGFC
LHAFTFLRDPWNWLDFSVIIMAYTTEFVDLGNVSALRTFRVLRALKTISVISGLKTIVGALIQSVKKLADVMVLTVFCLSVFALIGLQLFMGNLRHKCVRNFTALNGTNGSVEADGLVWESLDLYLSDPENYLLKNGTSDVLLCGNSSDAGTCPEGYRCLKAGENPDHGYTSFDSFAW
AFLALFRLMTQDCWERLYQQTLRSAGKIYMIFFMLVIFLGSFYLVNLILAVVAMAYEEQNQATIAETEEKEKRFQEAMEMLKKEHEALTIRGVDTVSRSSLEMSPLAPVNSHERRSKRRKRMSSGTEECGEDRLPKSDSEDGPRAMNHLSLTRGLSRTSMKPRSSRGSIFTFRRRDL
GSEADFADDENSTAGESESHHTSLLVPWPLRRTSAQGQPSPGTSAPGHALHGKKNSTVDCNGVVSLLGAGDPEATSPGSHLLRPVMLEHPPDTTTPSEEPGGPQMLTSQAPCVDGFEEPGARQRALSAVSVLTSALEELEESRHKCPPCWNRLAQRYLIWECCPLWMSIKQGV
KLVVMDPFTDLTITMCIVLNTLFMALEHYNMTSEFEEMLQVGNLVFTGIFTAEMTFKIIALDPYYYFQQGWNIFDSIIVILSLMELGLSRMSNLSVLRSFRLLRVFKLAKSWPTLNTLIKIIGNSVGALGNLTLVLAIIVFIFAVVGMQLFGKNYSELRDSDSGLLPRWHMMDFFHAFLIIF
RILCGEWIETMWDCMEVSGQSLCLLVFLLVMVIGNLVVLNLFLALLLSSFSADNLTAPDEDREMNNLQLALARIQRGLRFVKRTTWDFCCGLLRQRPQKPAALAAQGQLPSCIATPYSPPPPETEKVPPTRKETRFEEGEQPGQGTPGDPEPVCVPIAVAESDTDDQEEDEENSLG
TEEESSKQQESQPVSGGPEAPPDSRTWSQVSATASSEAEASASQADWRQQWKAEPQAPGCGETPEDSCSEGSTADMTNTAELLEQIPDLGQDVKDPEDCFTEGCVRRCPCCAVDTTQAPGKVWWRLRKTCYHIVEHSWFETFIIFMILLSSGALAFEDIYLEERKTIKVLLEYAD
KMFTYVFVLEMLLKWVAYGFKKYFTNAWCWLDFLIVDVSLVSLVANTLGFAEMGPIKSLRTLRALRPLRALSRFEGMRVVVNALVGAIPSIMNVLLVCLIFWLIFSIMGVNLFAGKFGRCINQTEGDLPLNYTIVNNKSQCESLNLTGELYWTKVKVNFDNVGAGYLALLQVATFKG
WMDIMYAAVDSRGYEEQPQWEYNLYMYIYFVIFIIFGSFFTLNLFIGVIIDNFNQQKKKLGGQDIFMTEEQKKYYNAMKKLGSKKPQKPIPRPLNKYQGFIFDIVTKQAFDVTIMFLICLNMVTMMVETDDQSPEKINILAKINLLFVAIFTGECIVKLAALRHYYFTNSWNIFDFVVVI
LSIVGTVLSDIIQKYFFSPTLFRVIRLARIGRILRLIRGAKGIRTLLFALMMSLPALFNIGLLLFLVMFIYSIFGMANFAYVKWEAGIDDMFNFQTFANSMLCLFQITTSAGWDGLLSPILNTGPPYCDPTLPNSNGSRGDCGSPAVGILFFTTYIIISFLIVVNMYIAIILENFSVATEESTEPLSE
DDFDMFYEIWEKFDPEATQFIEYSVLSDFADALSEPLRIAKPNQISLINMDLPMVSGDRIHCMDILFAFTKRVLGESGEMDALKIQMEEKFMAANPSKISYEPITTTLRRKHEEVSAMVIQRAFRRHLLQRSLKHASFLFRQQAGSGLSEEDAPEREGLIAYVMSENFSRPLGPPSSSSI
SSTSFPPSYDSVTRATSDNLQVRGSDYSHSEDLADFPPSPDRDRESIV

b

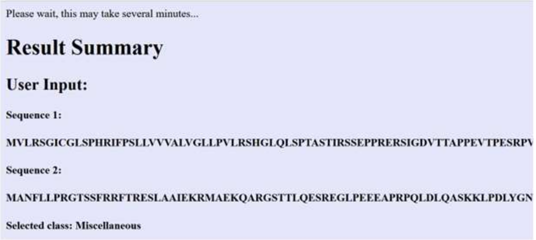

c

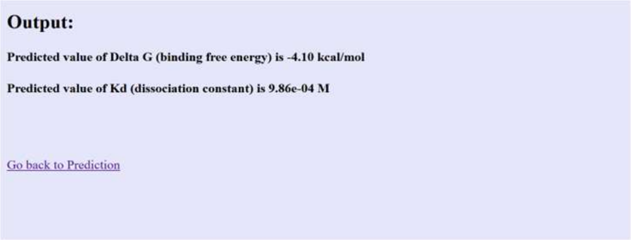

d

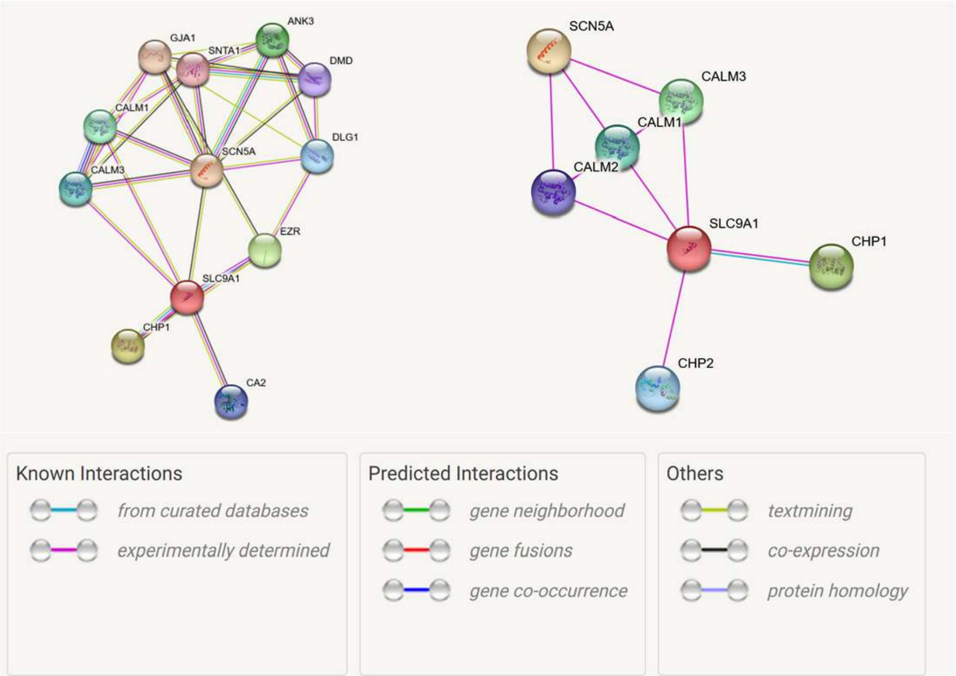

Figure S4. ***In silico*** **analysis for the prediction of NHE-1-Na$_V$1.5 proteins interaction**. **(a)** Amino acid sequence of NHE-1 (Uniprot ID: P19634-1) and Na$_V$1.5 (Uni-prot ID: Q14524-1) used as inputs for the online tool Protein-Protein Affinity Predictor (PPA-Pred2, https://www.iitm.ac.in/bioinfo/PPA_Pred/index.html). **(b)** screenshot of the server with the amino acid sequences of the proteins of interest. **(c)** Results showed a predicted value of binding free energy, ΔG = –4.10 kcal/mol and a dissociation constant, Kd = 9.86 x 10^{-04} M suggesting that the interaction between the two proteins has a low but real probability of occurring. **(d)** Identified networks of protein-protein interactions involving Na$_V$1.5 sodium channels and the NHE-1 exchanger in the database Search Tool for Recurring Instances of Neighbouring Genes (STRING, https://string-db.org/cgi/input?sessionId=bWoctneOwNlx&input_page_show_search=on). Na$_V$1.5 (SCN5A) and NHE-1 (SLC9A1) act as interaction nodes with multiple proteins including several members of the calmodulin family.
