# Supplementary material for: Voltage-Gated Sodium Channel NaV1.5 Controls NHE−1−Dependent Invasive Properties in Colon Cancer Cells
Source: Cancers (Basel). 2022 Dec 22;15(1):46. doi: 10.3390/cancers15010046 (PMC9817685; doi:10.3390/cancers15010046)
Supplement: Supplementary file 1 [file cancers-15-00046-s001.zip › Figure S5 Time course of inhibition of Nav1.5 currents by compound 4.pdf]

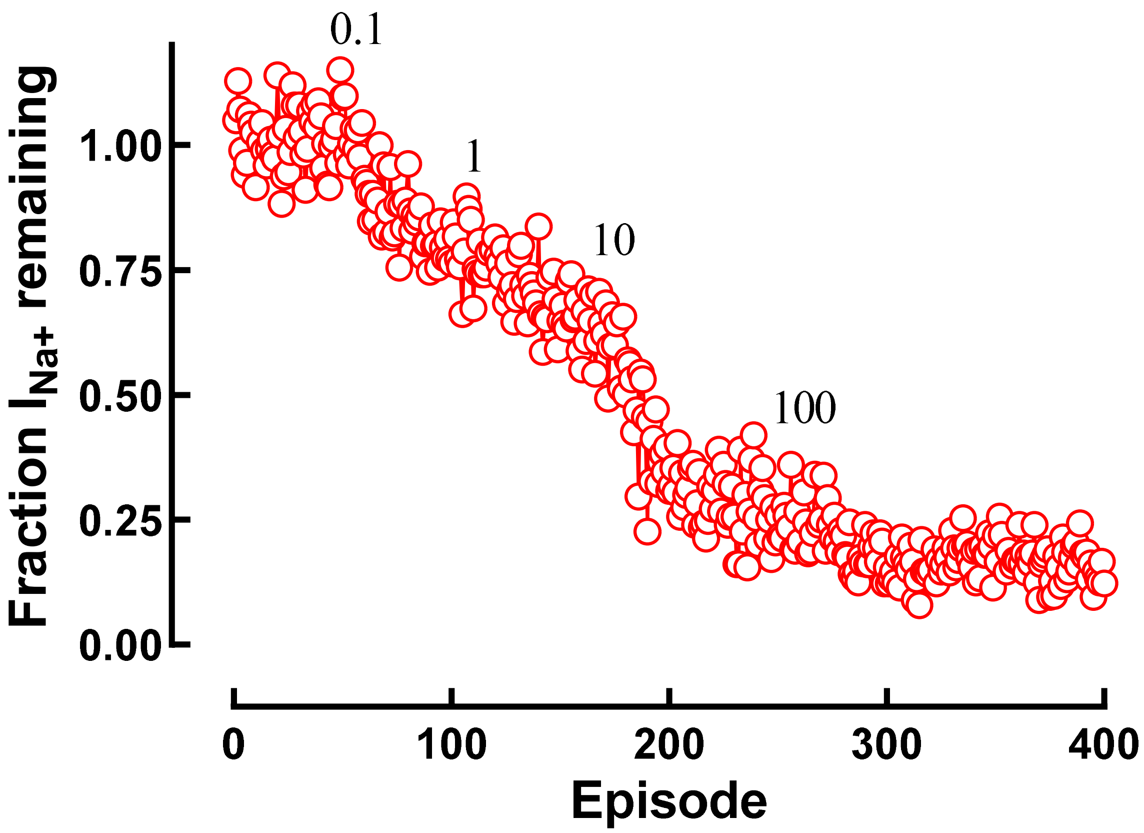

Figure S5. Time course of inhibition of $Na_V1.5$ currents by compound 4. Peak currents were normalized to control amplitude (before compound 4 exposure), defined as the fraction of I_{Na+} remaining. Each red circle means the amplitude of a normalized sodium current trace. The solid blue line connects the symbols. After perfusion of the lowest concentration of compound 4, it was waited until a steady state blockage was obtained before proceeding to the next concentration and so on. Compounds 4 was poorly washed out, exhibiting a recovery of the original sodium current < 20%.
