# Supplementary material for: Voltage-Gated Sodium Channel NaV1.5 Controls NHE−1−Dependent Invasive Properties in Colon Cancer Cells
Source: Cancers (Basel). 2022 Dec 22;15(1):46. doi: 10.3390/cancers15010046 (PMC9817685; doi:10.3390/cancers15010046)
Supplement: Supplementary file 1 [file cancers-15-00046-s001.zip › Figure S6 Small-molecule Nav1.5 inhibitors reduce the number of invasive cells in 2D invasion experiments.pdf]

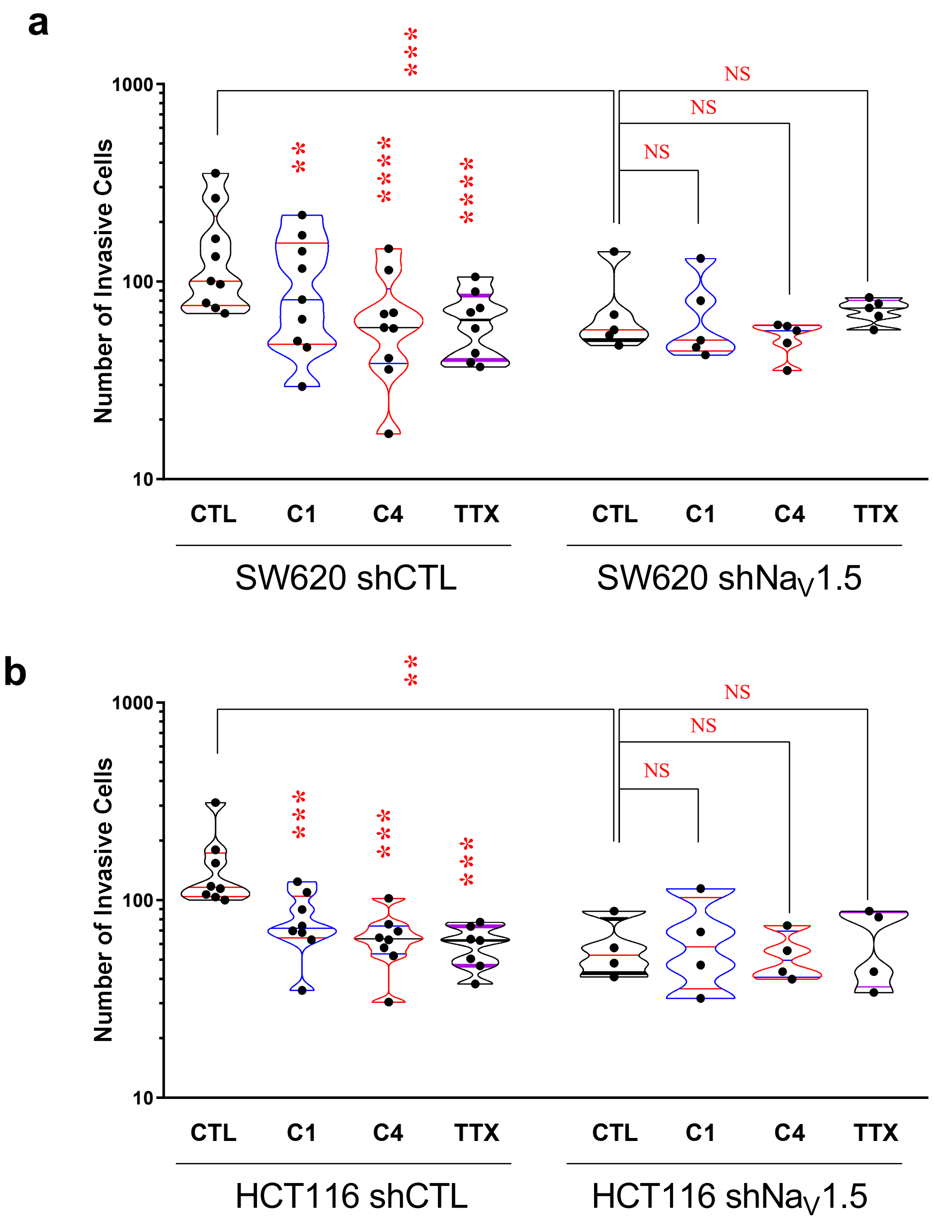

Figure S6. Small-molecule $Na_V1.5$ inhibitors reduce the number of invasive cells in 2D invasion experiments. **(a), (b),** Summary of SW620 and HCT116 colon cancer cell invasiveness studies performed on Matrigel-coated (0.3 mg/ml) invasion inserts in control condition (0.1% DMSO), in presence of Compound 1 (1μM), Compound 4 (1μM) or TTX (30μM). Results from 6-9 independent experiments and are presented as the row number of invasive cells that were counted for each condition. SW620 and HCT116 cancer cells stably expressing a short hairpin RNA targeting *SCN5A* gene expression ($shNa_V1.5$) were also treated under the same conditions. **$p < 0.01$; ***$p < 0.001$, ****$p < 0.0001$ Mann–Whitney rank sum test.
