# Supplementary material for: Voltage-Gated Sodium Channel NaV1.5 Controls NHE−1−Dependent Invasive Properties in Colon Cancer Cells
Source: Cancers (Basel). 2022 Dec 22;15(1):46. doi: 10.3390/cancers15010046 (PMC9817685; doi:10.3390/cancers15010046)
Supplement: Supplementary file 1 [file cancers-15-00046-s001.zip › Table S1 Anatomical distribution and staging of human colon cancer biopsies.pdf]

Table S1. Anatomical distribution and staging of human colon cancer biopsies

	Male	Female	Total
Samples	*76*	*60*	*136*
Tumor location			
Left	*12*	*2*	*14*
Sigmoid	*35*	*20*	*55*
Right	*18*	*25*	*43*
Caecum	*11*	*13*	*24*
TNM Staging			
I	*13*	*8*	*21*
II	*32*	*26*	*58*
III	*25*	*18*	*43*
IV	*6*	*6*	*12*
