# Supplementary material for: Voltage-Gated Sodium Channel NaV1.5 Controls NHE−1−Dependent Invasive Properties in Colon Cancer Cells
Source: Cancers (Basel). 2022 Dec 22;15(1):46. doi: 10.3390/cancers15010046 (PMC9817685; doi:10.3390/cancers15010046)
Supplement: Supplementary file 1 [file cancers-15-00046-s001.zip › Table S2 qPCR primers.pdf]

Table S2. Validated primers for qPCR experiments on colorectal cancer biopsies and cancer cell lines

Gene	Protein	Forward 5′ → 3′	Reverse 5′→ 3′	Product size (bp)
SCN1A	Nav1.1	TTCATGGCTTCCAATCCTTC	TAGCCCCACCTTTGATTTTG	178
SCN2A	Nav1.2	GCCAGCTTATCAATCCCAAA	TCTTCTGCAATGCGTTGTTC	192
SCN3A	Nav1.3	CAAAGGGAAGATCTGGTGGA	AAAGGCCAATGCACCACTAC	115
SCN4A	Nav1.4	TCAACAACCCCTACCTGACC	ACGGACGAGTTCCCATCATA	148
SCN5A	Nav1.5	CACGCGTTCACTTTCCTTC	CATCAGCCAGCTTCTTCACA	208
SCN8A	Nav1.6	CGCCTTATGACCCAGGACTA	GTGCCTCTTCCTGTTGCTTC	247
SCN9A	Nav1.7	GGCTCCTTGTTTTCTGCAAG	TGGCTTGGCTGATGTTACTG	196
SCN10A	Nav1.8	ACCTGGTGGTGCTTAACCTG	TGCTGAAGAAGCTGCAAAGA	168
SCN11A	Nav1.9	CTGTGGTCCTGGTCATTGTG	TGCATTCGCTTCTTGCATAC	233
HPRT1	Hprt1	TGCGCTATGAGAATGAGGTG	GAAGAAGAGCAGGCGGTAGA	176
PPIA	PPIA	AGGAGATGTTCCTCCAGTTCC	GCACGTTTCTCAGCATCACC	123
SLC9A1	NHE1	GAGGGCGGTAAAGATTTCCT	AGAGGCCAGAGTCGTTCAGA	154
SLC9A2	NHE2	GAAGTCTGACCCCAAGGTGA	CACATGGCAGGTGTATTTGC	139
SLC9A3	NHE3	TTGCTGACCTGCTGGATTAC	TATGTCCCCTGTTGACTGGT	119
SLC9A4	NHE4	ACCGCCGAGGAAAACCGTGTA	TGCTGTCTTTGGGACCTTGTCTGC	129
