# Supplementary material for: Voltage-Gated Sodium Channel NaV1.5 Controls NHE−1−Dependent Invasive Properties in Colon Cancer Cells
Source: Cancers (Basel). 2022 Dec 22;15(1):46. doi: 10.3390/cancers15010046 (PMC9817685; doi:10.3390/cancers15010046)
Supplement: Supplementary file 1 [file cancers-15-00046-s001.zip › Table S3 F-Medium composition.pdf]

Table S3. Composition of F-Medium for the establishment of tissue explants and colon derived primary cultures

Component	Final concentration
Fetal bovine serum (Gibco cat. No. 16140-071)	10%
L-Glutamine (Gibco cat. No. 25030-081)	1%
Penicillin/Streptomycin (Gibco cat. No.15140-122)	1%
Hydrocortisone (Sigma Aldrich cat. No. H-0888)	25 ng/ml
Epidermal growth factor (Life Technologies cat. No. PHG0313)	0.125 ng/ml
Insulin (Sigma Aldrich cat. No. I-5500)	5 μg/ml
Amphotericin B (Fisher Scientific cat. No. BP264550)	250 ng/ml
Gentamicin (Gibco cat. No. 15710-064)	10 μg/ml
Cholera Toxin (Sigma-Aldrich cat. No. C-3012)	0.1 nM
Y27632, ROCK inhibitor (Enzo, cat. No. 270-333M025)	10 μM

F-Medium was prepared using DMEM (Gibco cat. No. 11965-092) and F-12 nutrient mix (Gibco cat. No. 11765-054) (1:1)
