# Supplementary material for: Voltage-Gated Sodium Channel NaV1.5 Controls NHE−1−Dependent Invasive Properties in Colon Cancer Cells
Source: Cancers (Basel). 2022 Dec 22;15(1):46. doi: 10.3390/cancers15010046 (PMC9817685; doi:10.3390/cancers15010046)
Supplement: Supplementary file 1 [file cancers-15-00046-s001.zip › Table S4 Sets of primary antibodies used for colocalization analysis of NaV15 and NHE1 in colon cancer cells.pdf]

Table S4. Sets of primary antibodies used for colocalization analysis of Nav1.5 and NHE-1 in colon cancer cells

Primary antibodies set	Antibodies	Immunogen peptide	Epitope localization in the protein	Detected by epifluorescence with
No.1	Anti-Nav1.5 produced in rabbit Sigma-Aldrich Ref. S0819	Residues 439-511	Intracellular loop between S6 DI – S1 DII	Alexa Fluor 647-coupled secondary antibody
	Anti-NHE-1 produced in mouse Santa Cruz Biotechnologies Ref. sc-136239	Residues 682-801	located at the carboxyl-terminus	Alexa Fluor 488-coupled secondary antibody
No.2	Anti-Nav1.5 produced in mouse Santa Cruz Biotechnologies Ref. sc-271255	Residues 971 - 1140	Intracellular loop between S6 DII – S1 DIII	Alexa Fluor 488-coupled secondary antibody
	Anti-NHE-1 produced in rabbit Abcam Ref. ab67314	Residues 490 - 540	Intracellular region of M12	Alexa Fluor 647-coupled secondary antibody
