# Supplementary figures and images for: Voltage-Gated Sodium Channel NaV1.5 Controls NHE−1−Dependent Invasive Properties in Colon Cancer Cells
Source: Cancers (Basel). 2022 Dec 22;15(1):46. doi: 10.3390/cancers15010046 (PMC9817685; doi:10.3390/cancers15010046)

# Disease Free Survival

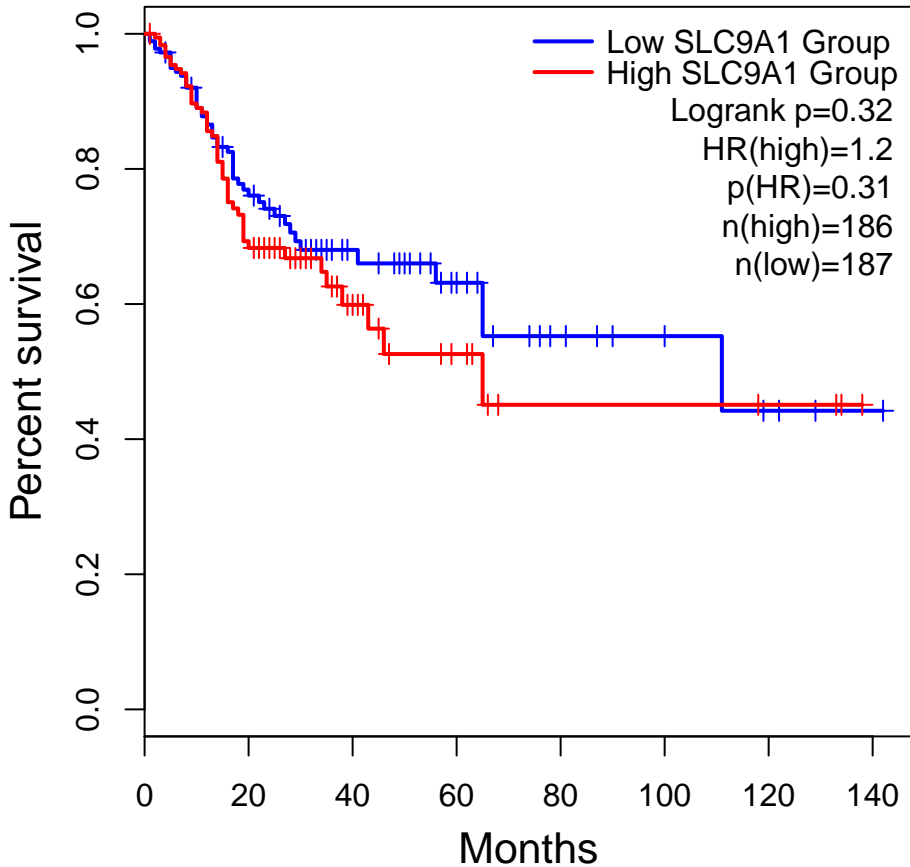

Supplement: Supplementary file 1 [file cancers-15-00046-s001.zip › Figure S2 SLC9A1-Disease Free Survival.pdf]
